# Molecular and clinical characterization of *PTRF* in glioma via 1,022 samples

**DOI:** 10.1186/s12885-023-11001-2

**Published:** 2023-06-16

**Authors:** Si Sun, Changlin Yang, Kuanyu Wang, Ruoyu Huang, Ke-nan Zhang, Yanwei Liu, Zhi Cao, Zheng Zhao, Tao Jiang

**Affiliations:** 1 Beijing Neurosurgical Institute, Capital Medical University, Beijing, 100070 China; 2 Department of Neurosurgery, Beijing Tongren Hospital, Capital Medical University, Beijing, 100730 China; 3 Department of Radiotherapy, Beijing Tiantan Hospital, Capital Medical University, Beijing, 100070 China; 4 Department of Neurosurgery, The Fourth Affiliated Hospital of China Medical University, Shenyang, 110032 China; 5 Chinese Glioma Genome Atlas Network and Asian Glioma Genome Atlas Network, Beijing, 100070 China; 6 Department of Neurosurgery, Beijing Tiantan Hospital, Capital Medical University, Beijing, 100070 China; 7 Center of Brain Tumor, Beijing Institute for Brain Disorders, Beijing, 100069 China; 8 China National Clinical Research Center for Neurological Diseases, Beijing, 100070 China; 9 Research Unit of Accurate Diagnosis, Treatment, and Translational Medicine of Brain Tumors, Chinese Academy of Medical Sciences, Beijing, 100070 China

**Keywords:** *PTRF*, Glioma, Prognosis, Immune response

## Abstract

Polymerase I and transcript release factor (*PTRF*) plays a role in the regulation of gene expression and the release of RNA transcripts during transcription, which have been associated with various human diseases. However, the role of *PTRF* in glioma remains unclear. In this study, RNA sequencing (RNA-seq) data (*n* = 1022 cases) and ﻿whole-exome sequencing (WES) data (*n* = 286 cases) were used to characterize the *PTRF* expression features. ﻿Gene ontology (GO) functional enrichment analysis was used to assess the biological implication of changes in *PTRF* expression. As a result, the expression of *PTRF* was associated with ﻿malignant progression in gliomas. Meanwhile, somatic mutational profiles and copy number variations (CNV) revealed the glioma subtypes classified by *PTRF* expression showed distinct genomic alteration. Furthermore, GO functional enrichment analysis suggested that *PTRF* expression was associated with cell migration and angiogenesis, particularly during an immune response. Survival analysis confirmed that a high expression of *PTRF* is associated with a poor prognosis. In summary, *PTRF* may be a valuable factor for the diagnosis and treatment target of glioma.

**Supplementary Information:**

The online version contains supplementary material available at 10.1186/s12885-023-11001-2.

## Introduction

Glioma is the most prevalent type of malignant brain tumor, with glioblastoma (GBM) being the most aggressive subtype [1–6]. Despite the use of standard treatment options such as surgery, radiation, and chemotherapy, patients with GBM typically have a median survival time of 14.6 months after diagnosis [7], and the 5-year survival rate ranges from 0.05% to 4.7% [8]. Identifying new biomarkers could help determine a more accurate prognosis for individuals with glioma and facilitate the development of improved treatment options [9–11].

PTRF, also known as cavin-1, is a protein that has been implicated in a number of human diseases and cancers. Jansa et al*.* revealed that PTRF interacted with RNA polymerase I and transcription termination factors to terminate transcription from yeast and mouse [12]. Similarly, Jansa and colleages suggested that PTRF is involved in the re-initiation of RNA polymerase I activity during gene transcription [13]. Previous studies have shown that overexpression of caveolin in a variety of tumours can enhance tumor cell migration, invasion and drug resistance, and is associated with poor patient survival [14–16]. Inder et al*.* reported that *PTRF* expression was implicated in the protein content of tumor-derived extracellular vesicles (proteasomes) and highlighted the potential of utilizing *PTRF*-mediated pathways to attenuate metastatic prostate cancer [17]. Recently, *PTRF* was also reported to be upregulated in patients with primary and recurrent GBM, and was considered to be a modulator of glioblastoma chemoresistance and immune responses [18–20]. However, the comprehensive transcriptome characterization and related functions of *PTRF* in glioma remain unclear.

In this study, we employed a combination of bulk genomic and transcriptomic profiling as well as scRNA-seq data to extensively investigate the involvement of *PTRF* in gliomas. Our findings shed light on the role of *PTRF* in glioma and have the potential to inform future clinical approaches for diagnosis and therapy of glioma.

## Materials and methods

### Sample and data collection

We obtained the RNA-seq data (*n* = 325 cases) and WES data (*n* = 286 cases) from the Chinese Glioma Genome Atlas (CGGA) database (http://cgga.org.cn) as a discovery set [21–23]. The matched clinical information includes *IDH* (*IDH1* and *IDH2*) mutation and chromosome 1p and 19q codeletion status, chemoradiotherapy information, et al. In addition, we also obtained the RNA-seq (*n* = 697 cases) and matched clinical data from the Cancer Genome Atlas (TCGA) (http://cancergenome.nih.gov) as a validation set. ﻿To explore the *PTRF*’s role in disease progression, we also obtained paired primary and recurrent glioma RNA-seq data (n = 132 cases) in the Glioma Longitudinal Analysis Consortium (GLASS) database [24] (http://www.synapse.org/glass). To explore the *PTRF* expression pattern in different histological regions, we obtained the Ivy data from the Ivy Glioblastoma Atlas Project–Allen Institute for Brain Science datasets database (http://glioblastoma.alleninstitute.org/, *n* = 270 cases) [25]. As our previous study [26], we obtain the scRNA-seq data of diffuse glioma patients [27]. The dataset comprises 6,863 tumor cells, 754 macrophages, 219 oligodendrocytes, and 94 T cells. For tumor cells, we also obtained annotations for four distinct cellular states, namely neural-progenitor-like (NPC-like), oligodendrocyte-progenitor-like (OPC-like), astrocyte-like (AC-like), and mesenchymal-like (MES-like) states. This study was approved by the Ethics Committee of Beijing Tiantan Hospital, Capital Medical University, Beijing, China.

### Immunohistochemistry analysis

The selected glioma samples were collected from the CGGA tissue bank and were supervised by the Beijing Tiantan Hospital Institutional Review Board (KY 2019–143-02). IHC analysis was performed as previously reported [28]. Briefly, brain tumor sections were incubated with the PTRF (1:1000, 69036S, Cell Signaling Technology) antibody overnight at room temperature. Then, the stained sections were scored by two independent pathologists. The stain-ing intensity was 0–3 points: 0 (negative), 1 (weak), 2 (moderate) and 3 (strong). The extent of staining reflected the percentage of positive cells: 0 (< 5%),1 (6%-25%),2 (26%-50%), 3 (51%-75%)and 4 (> 75%). The staining index was defined as the product of staining intensity and staining extent.

### Statistical analysis

R (v4.2.2) software was used as the main tool for the statistical analysis and generation of figures. Multivariable Cox proportional hazard model was performed using R package survival *coxph* function. We utilized the R package ggplot2 to generate the visualizations presented in this study. Other figures were generated by several R packages, such as pheatmap, pROC, circlize, rms, ggfortify and corrgram. *P* < 0.05 was considered to be statistically significant.

### CGGA CNV data analysis

WES data were mapped to the human reference genome (hg19) using the Burrows-Wheeler Aligner (BWA) tool [29] with default parameters. Then, SAM tools [30] and Picard (http://broadinstitute.github.io/picard/) were used to sort the reads by coordinates and mark duplicates. Next, we used the CNVkit software [31] to estimate the CAN status of well-known driver genes in gliomas, such as *PTEN*, *PDGFRA*, *EGFR*, and *CDKN2A/B*. In this study, a copy number gain is identified as log_2_ (ratio) larger than 0.5, while a copy number loss is identified as log_2_ (ratio) less than -1.

### Gene ontology (GO) analysis

The Pearson correlation analysis of *PTRF* and other genes in expression profiles was performed with the CGGA and TCGA datasets. To detect the biological processes that were related to *PTRF* expression in glioma pathology, positively or negatively correlated genes (R > 0.6 or R < -0.6; *P* < 0.05) were analyzed using the Database for Annotation, Visualization and Integrated Discovery (URL:http: //david.abcc.ncifcrf.gov) online tool. GO Functional enrichment maps were generated using the WEB-based Gene Set Analysis Toolkit (URL: http://www.webgestalt.org).

## Results

### PRTF expression is associated with malignant progression in glioma patients

**Fig. 1:**
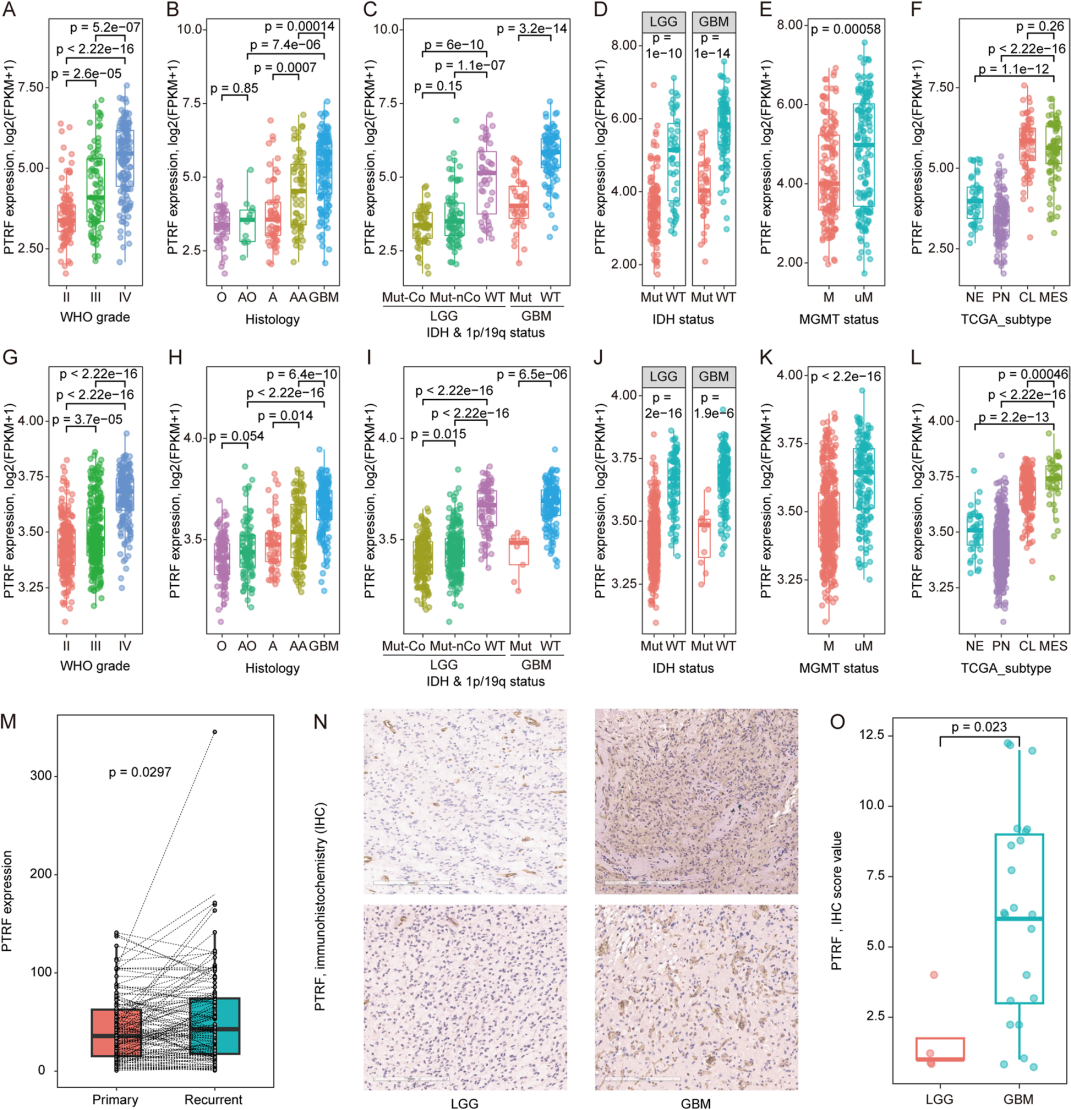
*PTRF* expression pattern in glioma. **A** and **G** WHO grade; **B** and **H** histology; **C** and (**I**) 2016 WHO classification; **D** and **J** *IDH* mutation status in LGGs and GBMs; **E** and **K** *MGMT* promoter methylation status; **F** and **L** TCGA subtype. **A-F** for CGGA dataset and **G-L** for TCGA dataset. **M** Traced lines indicate the *PTRF* expression changes between primary and paired recurrent glioma. **N** Representative immunohistochemistry (IHC) of PTRF in low-grade glioma and high-grade glioma tissues. **O** Dot plots of immunohistochemistry in LGG and GBM

The expression of *PTRF* in 325 samples was analyzed with RNA sequencing data from the CGGA database. WHO grade IV GBM samples exhibited a significantly higher expression of *PTRF* compared with grades II and III (all *p* ≤ 2.6e-5, Fig. 1A). Furthermore, our results suggested that GBMs show higher *PTRF* expression than other histology (all *p* ≤ 1.4e-4, Fig. 1B). It is well-known that *IDH* mutation status is closely related to the malignant progression of glioma [32]. In LGG, we explored the expression of *PTRF* was highest in ﻿*IDH* wild-type while the lowest was in *IDH* mutation and 1p/19q co-deletion subtype (all *p* ≤ 1.1e-7, Fig. 1C). Both LGG and GBM exhibited a downregulation in *PTRF* expression with an *IDH* mutation when compared with *IDH* wild-type gliomas (all *p* ≤ 1e-10, Fig. 1D). In addition, MGMT promoter unmethylation type shows a significantly higher expression of *PTRF*, indicating that higher *PTRF* expression is closely related to glioma ﻿temozolomide (TMZ) therapy resistance (*p* ≤ 6e-4, Fig. 1E). To investigate the molecular association between *PTRF* and glioma, *PTRF* expression and molecular subtypes were defined with the TCGA subtype classification system [33]. The mesenchymal and classical subtype displayed a higher expression of *PTRF* in the CGGA datasets (*p* ≤ 1.1e-12, Fig. 1F). These results were validated with the TCGA RNA sequencing dataset (Fig. 1G-L). Specially, the mesenchymal subtype showed significantly higher *PTRF* expression than other subtypes in the TGGA dataset. We also found that paired recurrent glioma exhibited a higher expression of *PTRF* compared to primary glioma in the GLASS database. (*p* = 0.0297, Fig. 1M, one-sided paired t-test). Consistently, the immunohistochemistry (IHC) of glioma patients showed that *PTRF* was higher expression in GBM patients than LGG patients (*p* = 0.023, Fig. 1N–O). Overall, these results suggested that *PTRF* is highly expressed in these malignant subtypes and may become a biomarker for malignant progression in glioma.

### Genomic alterations of *PTRF* expression subtype in glioma

**Fig. 2:**
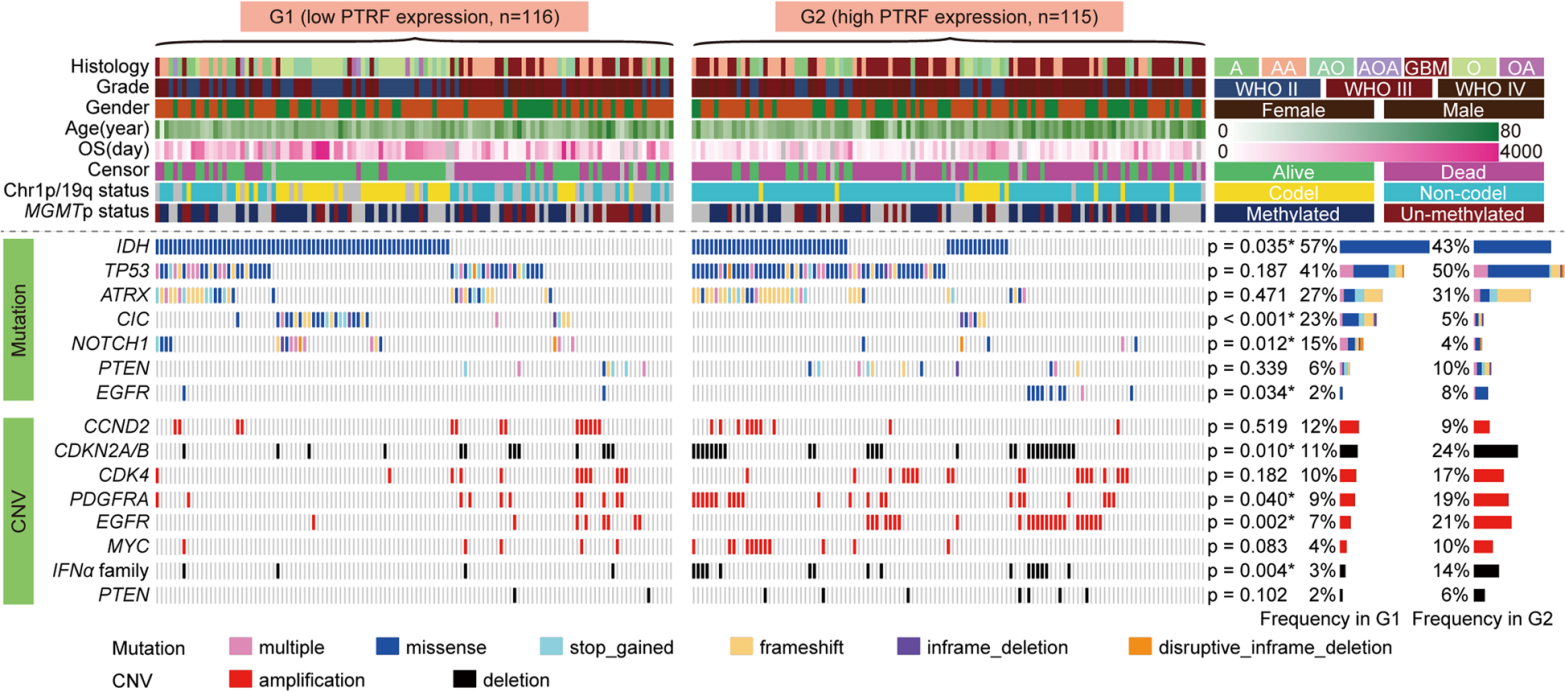
Mutational profile of glioma with high and low expression of *PTRF*

To explore the association between *PTRF* expression and genomic alteration in glioma, we analyzed the somatic mutations and copy number variations data from 231 samples having both RNA-seq and WES data in the CGGA dataset. Glioma samples were divided into G1 group (low expression) and G2 group (high expression) according to *PTRF* expression. Referring to previous research, we observed mutation frequency in *﻿IDH, TP53, ATRX, CIC, NOTCH1, PTEN,* and *EGFR* in this study (Fig. 2). Cases in G1 group were rich in *IDH, CIC*, and *NOTCH1* mutations that have been confirmed to be enriched in low grade glioma. On the contrary, *EGFR* mutations frequency in G2 group were higher than those in G1 group, suggesting that the high expression of *EGFR* may be related to the proliferation of glioma cells. In addition, we also explored the association between *PTRF* expression and copy number alterations. Consistent with high expression of *PTRF* in GBM, G2 group had a higher alteration frequency in *CDKN2A/B* deletion*, PDGFRA* amplification and *EGFR* amplification. According to previous reports, Interferon α plays an important role in anti-tumor immunity [34]﻿, we explored that Interferon α family deletion frequency was significantly higher in G2 group, suggesting that *PTRF* may be related to anti-tumor immunity mediated by Interferon α family. These results indicate that the glioma subtypes classified by *PTRF* expression shows distinct genomic alteration.

### *PTRF*-related biological processes in glioma

**Fig. 3:**
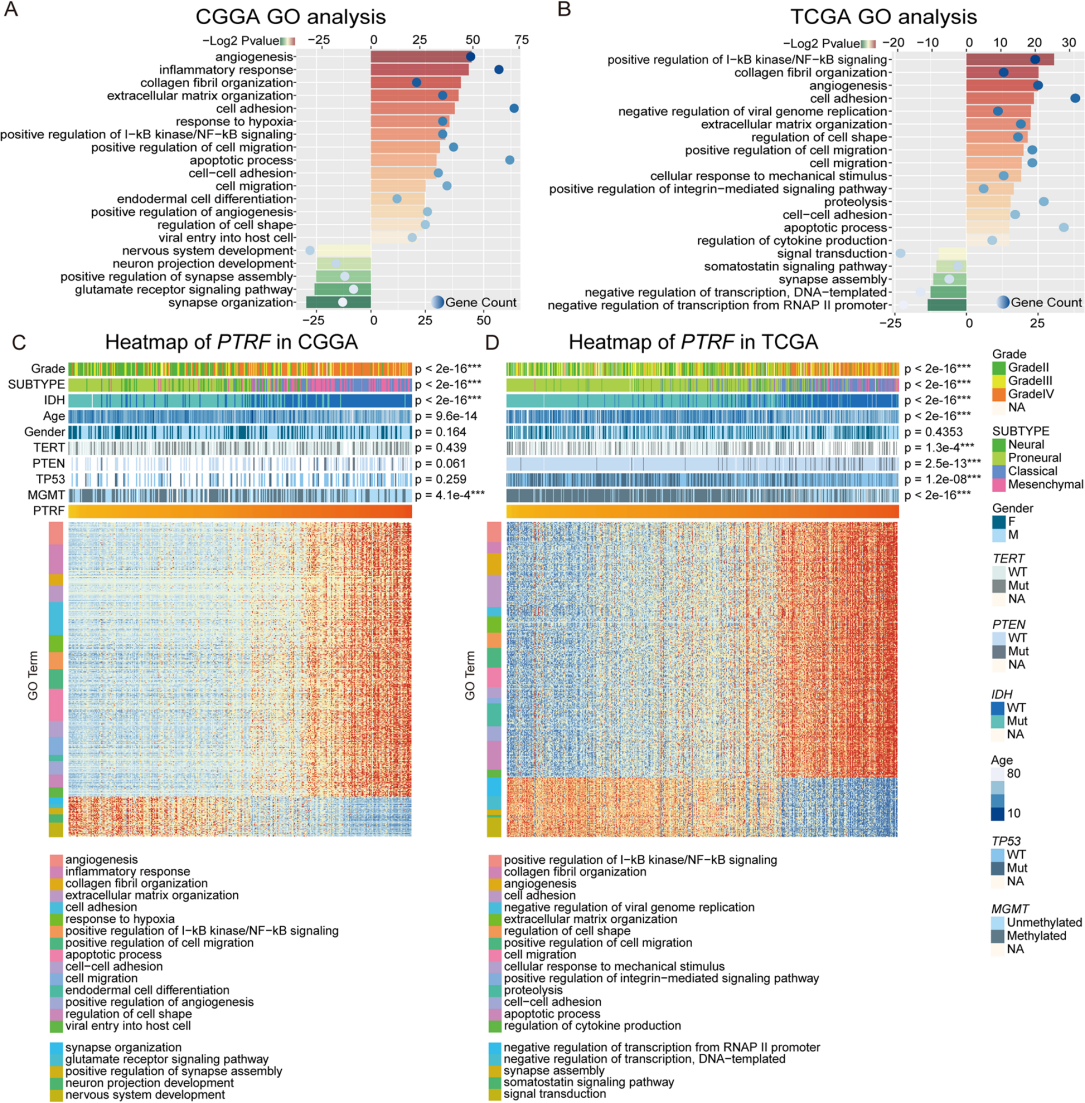
Gene ontology analysis of *PTRF* in glioma. **A** The gene set enrichment analysis of *PTRF* expression associated genes in CGGA dataset. **B** The gene set enrichment analysis of *PTRF* expression associated genes in TCGA dataset. **C** The heat map shows the expression pattern of *PTRF* expression associated genes in CGGA dataset. **D** The heat map shows the expression pattern of *PTRF* expression associated genes in TCGA dataset

 To investigate the relationship between the biological characteristics of glioma and *PRTF* expression, genes that exhibited a statistically significant correlation with *PTRF* expression (Pearson |R|> 0.6 and *p* < 0.05) were selected in CGGA (*n* = 325 cases) and TCGA (*n* = 697 cases) datasets. Two datasets were chosen for GO analysis with the DAVID online tool. A total of 20 gene ontology terms in two datasets were positively correlated with *PTRF* expression and involved in the *inflammatory response*, *cell migration*, *angiogenesis*, *cell adhesion*, *positive regulation of I-kappaB kinase/NF-kappaB signaling* (Fig. 3A-B). On the contrary, a total of 10 terms were found to be negatively correlated with *PTRF* expression, which was involved in neurological processes, such as nervous system development and function of synapse (Fig. 3A-B). The GO functional enrichment analyses were also drawn with WebGestalt (WEB-based Gene SeT AnaLysis Toolkit); biological adhesion, developmental processes, biological regulation, response to stimuli and cellular processes were highlighted (Figure S1 and 2). The selection criteria in these two cohorts were the ten with the lowest p-values, respectively. As previously mentioned in Fig. 1, grade, subtype, *IDH* mutation status and *MGMT* status were found to be associated with *PTRF* expression, we also found some molecular markers, such as *TERT*, *PTEN*, and *TP53* were associated with *PTRF* expression in TCGA, whereas gender was not found to have any correlation to *PTRF* expression (Fig. 3C-D).

### The role of *PTRF* in the immune response to glioma

**Fig. 4:**
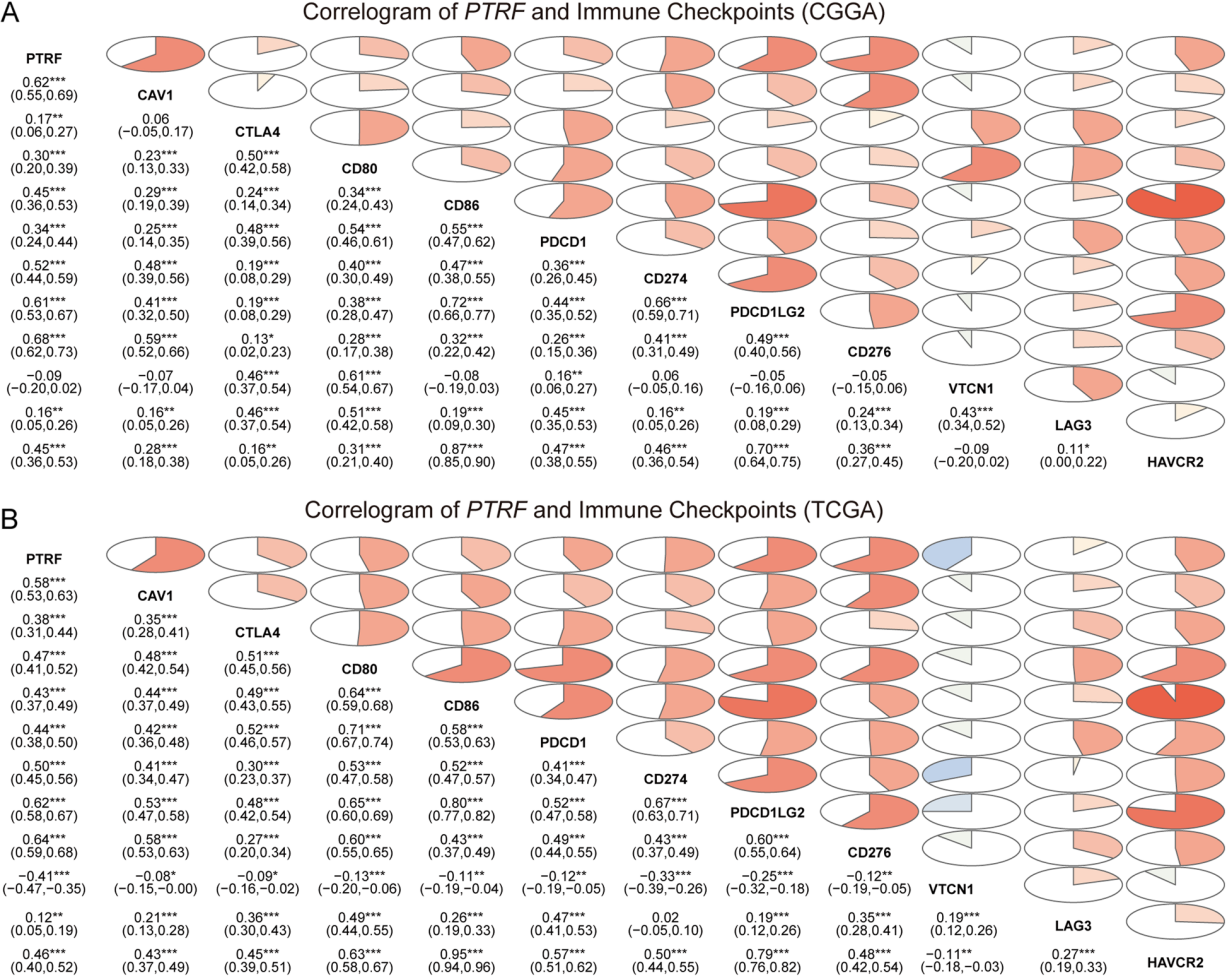
*PTRF*-related immune genes in CGGA and TCGA databases. **A** Correlogram of *PTRF* and Immune Checkpoints in CGGA dataset. **B** Correlogram of *PTRF* and Immune Checkpoints in TCGA dataset

**Fig. 5:**
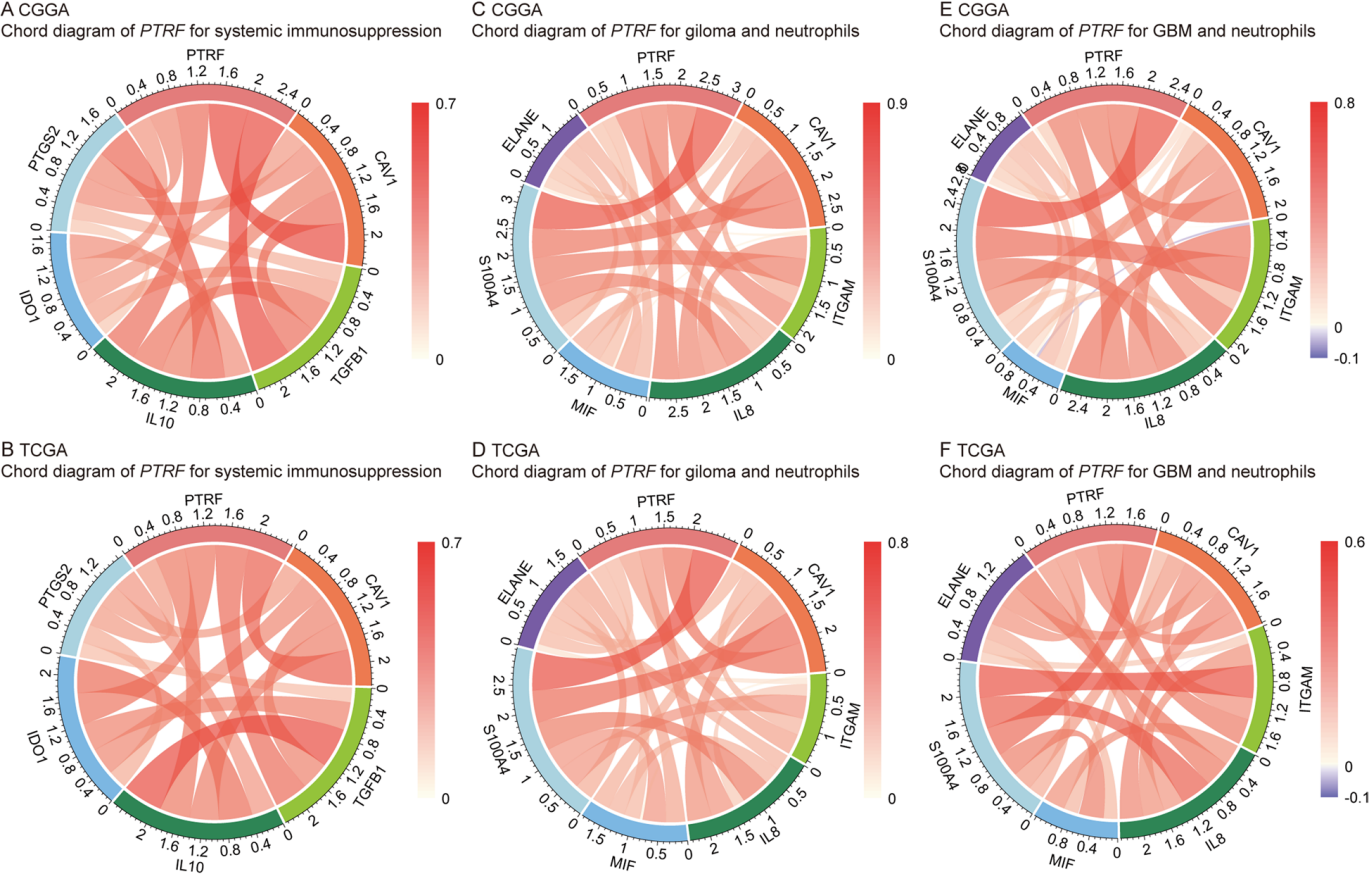
*PTRF*-related systemic immunosuppression and neutrophils in CGGA and TCGA databases. **A**-**B** *PTRF*-related systemic immunosuppression in CGGA and TCGA databases. **C**-**F** *PTRF*-related neutrophils in CGGA and TCGA databases

As shown in Fig. 3, *PTRF*-related biological functions constitute an important proportion of the immune and inflammatory response to glioma. Previous research reported that the blockade of immune checkpoints activated therapeutic anti-tumor immunity [35]. In the present study, immune checkpoint members, such as *CTLA4, CD80, CD86, PDCD1, CD274, PDCD1LG2, CD276, VTCN1, LAG3* and *HAVCR2*, were analyzed [36, 37]. Pearson’s correlation analysis was performed to examine the relationship between the expression of these genes and *PTRF* in both the CGGA and TCGA glioma datasets (Fig. 4). Caveolin-1, an indispensable component of caveolae formation alongside *PTRF*, was also included in this analysis. Correlograms revealed that the expression of *PTRF* and caveolin-1 was tightly associated with *PDCD1LG2, CD274, PDCD1* and *CD80*, indicating activation of *PD1* and *PD-L1* pathway. *HAVCR2* and *CD276* also showed a correlation with PTRF and caveolin-1. *VTCN1* expression was inconsistent. These results may be due to a suppressive effect on the T-cell related immune response. Overexpression of systemic immunosuppressive soluble factors by glioma cells, such as *PTGS2* (rate-limiting step catalyzed by COX-2), *TGFB1*, *IDO1*, and IL-10 [38], were also investigated at the transcriptional level (Fig. 5A-B). Several immune agents that interact with neutrophils and GBM were selected to be included in the analysis: *ITGAM, IL8* and *MIF, S100A4* and *ELANE* [38]. Chord diagrams were drawn of grade II-IV glioma and GBM from CGGA and TCGA datasets, respectively (Fig. 5C-F). The correlation of *PTRF* with these immune molecular markers revealed that *PTRF*, caveolin-1 and neutrophil activation were paralleled in GBM, which indicated a more aggressive phenotype. It may be inferred that *PTRF* and caveolin-1 within caveolae participate in the immune response to glioma.

### *PTRF* is highly expressed in microvascular proliferation (MVP) regions and MES tumor cells

**Fig. 6:**
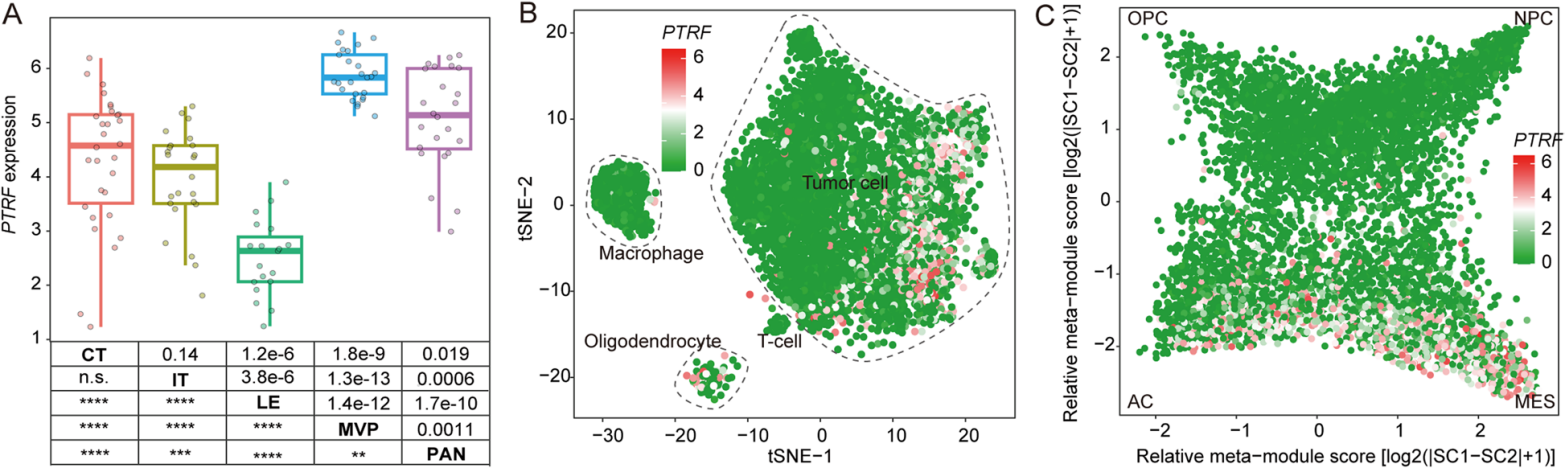
*PTRF* is highly expressed in microvascular proliferation (MVP) regions and MES tumor cells. **A** Expression of *PTRF* in different histological regions of GBM in Ivy dataset. **B** The single-cell data showed that *PTRF* was highly expressed in tumor cells. **C** Expression of *PTRF* in different glioma cellular states

To further explore *PTRF* expression in different histological regions of GBM, we collected anatomic transcriptional data in gliomas from Ivy Glioblastoma Atlas [25], including cellular tumor (CT), infiltrating tumor (IT), leading edge (LE), microvascular proliferation (MVP), and pseudopalisading cells around necrosis (PAN). We found that *PTRF* was significantly overexpressed in MVP region (Fig. 6A). This result was consistent with previous findings that *PTRF* is positively associated with angiogenesis in glioma. To further explore the expression of *PTRF* in the glioma environment, we collected single-cell transcriptomic data in glioblastoma from previous study [27]. We found that *PTRF* is highly expressed in tumor cell (Fig. 6B), especially glioma cells with MES cellular state (Fig. 6C), which contain a large subset of proliferating cells with an aggressive nature. In summary, we found that *PTRF* is highly expressed in microvascular proliferation (MVP) regions and MES tumor cells.

### *PTRF* predicts worse survival for glioma patients

**Fig. 7:**
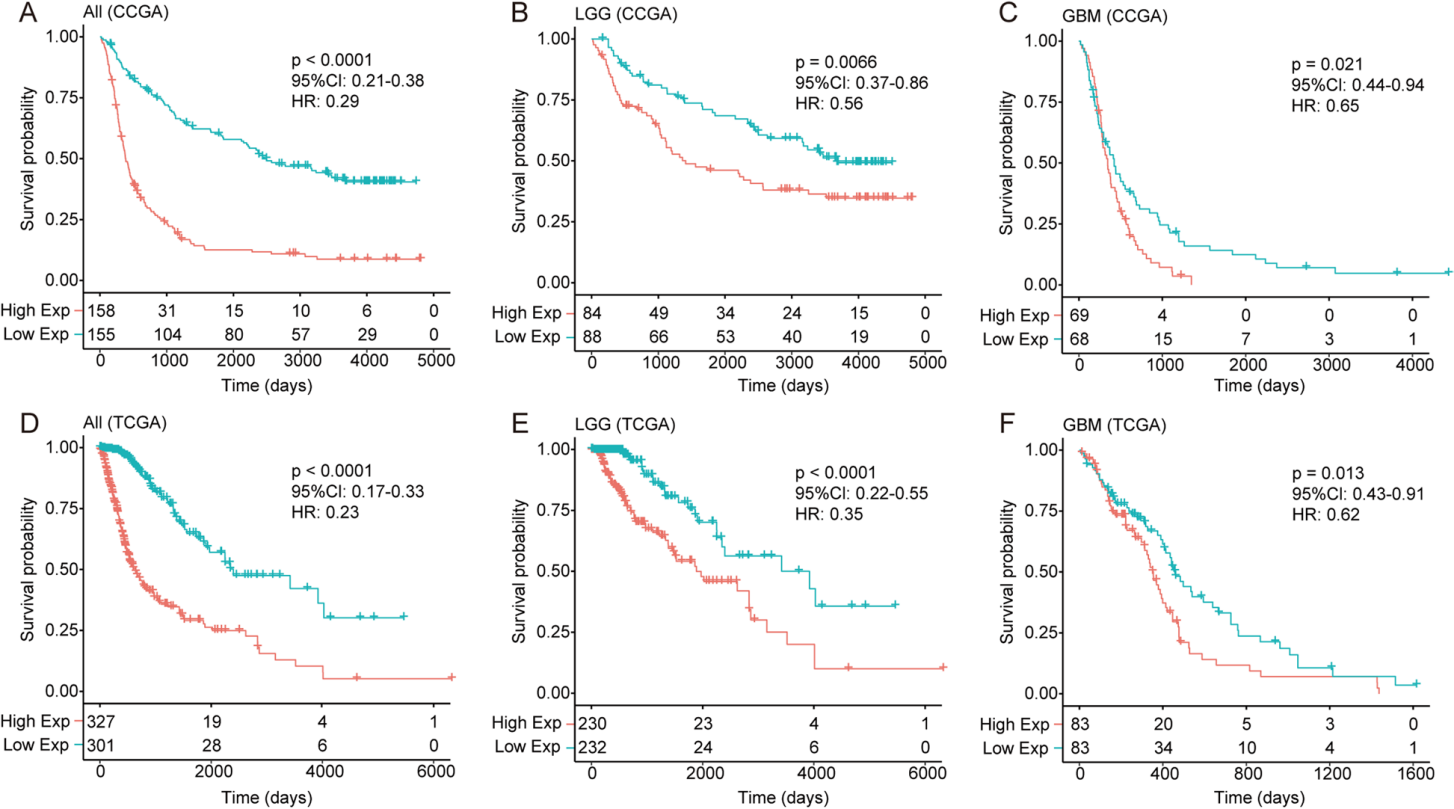
Survival analysis for *PTRF* in glioma patients. **A** Kaplan–Meier survival analysis of all grades of glioma patients in CGGA dataset based on *PTRF* expression. **B** Kaplan–Meier survival analysis of LGG patients in CGGA dataset based on *PTRF* expression. **C** Kaplan–Meier survival analysis of GBM patients in CGGA dataset based on *PTRF* expression. **D** Kaplan–Meier survival analysis of all grades of glioma patients in TCGA dataset based on *PTRF* expression. **E** Kaplan–Meier survival analysis of LGG patients in TCGA dataset based on *PTRF* expression. **F** Kaplan–Meier survival analysis of GBM patients in TCGA dataset based on *PTRF* expression

**Fig. 8:**
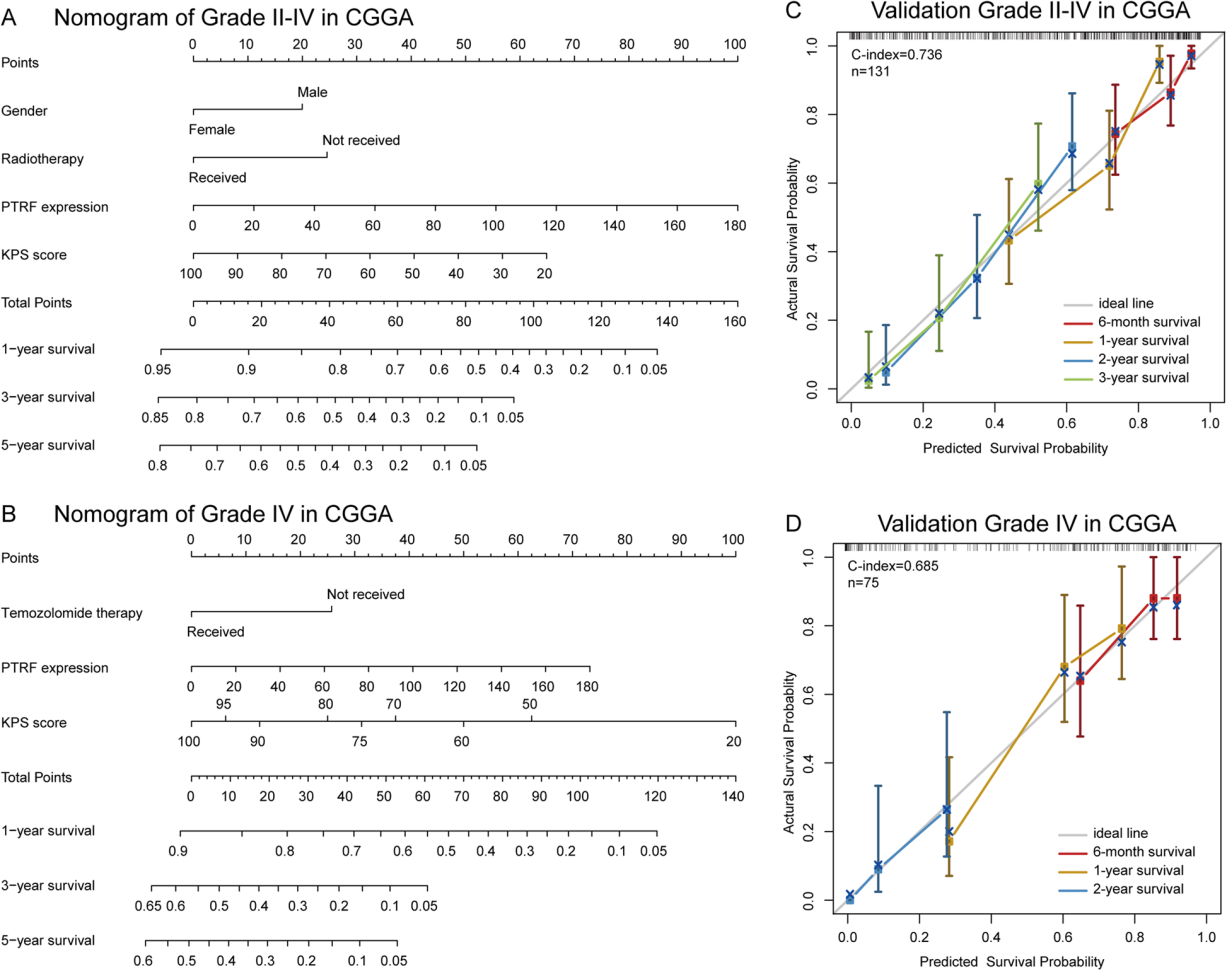
Nomogram for predicting 1-, 3- and 5-year mortality in association with *PTRF* expression and clinical data. **A** Nomogram of all grades of glioma patients in CGGA dataset. **B** Nomogram of GBM patients in CGGA dataset. **C** The calibration curve for predicting patient survival of all grades of glioma patients in CGGA dataset. **D** The calibration curve for predicting patient survival of GBM patients in CGGA dataset

As previously mentioned in Fig. 1, *PTRF* expression was consistent with the malignant progression and immune response in glioma. The prognostic value of *PTRF* was tested in ﻿both CGGA and TCGA databases, showing that patients with significantly shorter survival (all *p* < 0.05) exhibited a higher expression of *PTRF* (Fig. 7). As shown in Table 1, 131 events in grade II-IV glioma and 75 events in GBM were selected from the CGGA cohort with integrated data. The multivariate Cox regression analysis (backward stepwise) revealed that PTRF expression, gender, KPS score and radiotherapy were independent prognostic factors for the overall survival of patients with glioma grade II-IV. On the other hand, in the GBM group, *PTRF* expression, KPS score and TMZ therapy were shown to be independent prognostic factors by univariate and multivariate Cox regression analysis (Table 1). The established nomogram (Fig. 8A-B) illustrated that *PTRF* expression was the most prominent contributory component of grade II-IV groups, followed by KPS score, radiotherapy and gender. When analyzing the grade IV group, *PTRF* was found to contribute less compared with KPS score, but more compared with TMZ treatment. The concordance index (C-index) was calculated to evaluate the performance of the nomogram. The C-index of the nomogram for grade II-IV groups was 0.736, which was significantly higher than the constituting factors (*PTRF*, C-index = 0.68, *P* < 0.0001; KPS score, C-index = 0.689, *P* < 0.0001; radiotherapy C-index = 0.587, *P* < 0.0001; gender, C-index = 0.56, *P* = 0.013) (Fig. 8C). In the GBM group, the C-index (0.685) was worse, and the constituting factors were lower (*PTRF*, C-index = 0.555, *P* < 0.0.223; KPS score, C-index = 0.66, *P* < 0.0001; radiotherapy C-index = 0.618, *P* < 0.0001) (Fig. 8D). The C-index of *PTRF* expression showed no statistical significance, perhaps due to the small size of sample events in the GBM group. A calibration plot for the probability of survival at 0.5, 1 and 2 years in 2 event groups, then 3 years in the grade II-IV group, showed an accurate alignment between observation and prediction. Taken together, all these results suggest that *PTRF* may be an independent prognostic biomarker for glioma patients.

**Table 1:** The summary of clinicopathologic features and results of Cox regression analyses

Variable (Grade II-IV, *n* = 131)		Univariate					Multivariate				
**(Grade IV, *n* = 75)**		**HR**	**95% CI**			***P* value**	**HR**	**95% CI**			***P* value**
**Gender**	Female/Male(%)										
Grade II-IV	43/88 (67.2%)	0.577	0.365	-	0.914	0.019	0.540	0.315	-	0.926	0.025
Grade IV	22/53 (70.7%)	0.774	0.439	-	1.365	0.377					
**Age**	Mean/Range (Lo-Hi)										
Grade II-IV	45 (8–81)	1.027	1.006	-	1.048	0.010	1.012	0.989	-	1.035	0.306
Grade IV	47.8 (8–81)	0.994	0.972	-	1.017	0.623					
**KPS score**	Mean/Range (Lo-Hi)										
Grade II-IV	78.9 (20–100)	0.979	0.969	-	0.989	< 0.001	0.982	0.969	-	0.994	0.005
Grade IV	76.6 (20–100)	0.971	0.955	-	0.988	0.001	0.966	0.949	-	0.984	< 0.001
**PTRF expression**	Mean/Range (Lo-Hi)										
Grade II-IV	35.986 (2.610–163.403)	1.016	1.011	-	1.022	< 0.001	1.011	1.003	-	1.019	0.005
Grade IV	47.761 (5.665–163.403)	1.011	1.003	-	1.019	0.009	1.011	1.003	-	1.020	0.008
**IDH mutation status**	Mutated/WildType(%)										
Grade II-IV	52 (79 (60.3%)	0.298	0.188	-	0.471	< 0.001	0.651	0.344	-	1.231	0.186
Grade IV	14/61 (81.3%)	0.975	0.529	-	1.799	0.936					
**MGMT promotor status**	Methylated/Unmethylated(%)										
Grade II-IV	68/63 (48.1%)	0.585	0.388	-	0.880	0.010	0.820	0.522	-	1.288	0.389
Grade IV	34/41 (54.7%)	0.724	0.442	-	1.186	0.200					
**Temozolomide therapy**	Received/Not received(%)										
Grade II-IV	74/57 (43.5%)	1.406	0.921	-	2.144	0.114					
Grade IV	48/27 (36%)	0.517	0.312	-	0.859	0.011	0.495	0.295	-	0.832	0.008
**Radiotherapy**	Received/Not received(%)										
Grade II-IV	92/39 (52.7%)	0.550	0.358	-	0.844	0.006	0.511	0.325	-	0.803	0.004
Grade IV	50/25 (33.3%)	0.659	0.395	-	1.098	0.109					
**Vital status**	Alive/Dead(%)										
Grade II-IV	37/94 (71.8%)										
Grade IV	9/66 (88%)

## Discussion

Glioma is the most common primary malignant adult brain tumor, which has an unsatisfactory curative outcome with the traditional treatment of a combination of surgery, chemotherapy and radiotherapy [1–4, 7, 8]. *PTRF*, which is known as a regulator of transcription termination and an important component of caveolae alongside caveolin-1, is widely present in many mammalian cells [39]. *PTRF* and caveolin-1 downregulation is linked with other malignant tumors, such as prostate cancer, breast cancer and non-small-cell lung cancer [39, 40]. It was therefore unexpected that *PTRF* mRNA expression was observed to be higher according to the degree of malignancy in glioma [41]. Despite the unknown mechanism underlying the involvement of *PTRF* in the development of glioma, some articles refer to *PTRF*/caveolin-1/caveolae in association with GBM [41]. The present hypothesized that there is a similar link of *PTRF* with caveolins in glioma, as had been previously reported in other tumor cells [40]**.** In the present study, the role of *PTRF* in glioma biology was analyzed with GO analysis. CGGA and TCGA datasets were included, and a total of 1022 samples were enrolled in the analysis. It was revealed that high *PTRF* mRNA expression is linked to high grade gliomas, which suggests that high *PTRF* mRNA expression level is accompanied by a malignant biological phenotype. *IDH* wildtype was also consistent with *PTRF* upregulation and *PTRF* expression was lowest in *IDH*-mutated low-grade gliomas, suggesting that high *PTRF* mRNA expression level was associated with *IDH*-related immune response and a poor prognosis. Additionally, *PTRF* is highly expressed in mesenchymal subtype, a microsatellite-stable with epithelial-to-mesenchymal transition phenotype, which has the worst prognosis among all the four TCGA subtypes. This suggests that the function of *PTRF* is different in glioma tissue compared to other tumor types. Another study also reported a positive correlation between *PTRF* and WHO grade, recurrence and chemotherapy resistance of glioma [20]. In heatmaps of *PTRF*, the expression of *PTRF* is positively associated with angiogenesis and cell migration in glioma tissue.

Interestingly, it was also discovered in the present study that the inflammatory response, cell adhesion, positive regulation of I-kappaB kinase/NF-kappaB signaling are related to the expression of *PTRF* with GO functional analysis. The data revealed that *PTRF* plays a key role in the immune response between glioma cells and infiltrating immune cells. Glioma cells use multiple strategies to suppress immune responses, such as downregulating their own MHC-I complexes, as well as increasing the expression of immune checkpoint regulators, such as PD-1 and PD-L1 [42]. In this study, we revealed that the upregulation of *PTRF* and caveolin-1 is accompanied by an increase in the expression of immune checkpoint regulators. Many are initiated by ligand-receptor interactions, which may be located in caveolae [43]. The blockade of immune checkpoints is a promising approach to anti-tumor immunotherapy [35]. Consistently, *PTRF* and caveolin-1 are associated with the promotion of inflammatory signals, likely through the localization of inflammatory receptors to the caveolae signaling platform [44]. Some studies have concluded that *PTRF* plays a crucial role in regulating the function of macrophages after poly-microbial infection in mice [45], but no study has directly investigated the role of *PTRF* in inflammation. We found that *PTRF* and caveolin-1 were expressed synergistically with systemic immunosuppressive soluble factors like *TGFB1*, *IL10*, *IDO1*, and *PTGS2*. Almost all these factors decrease the pro-inflammatory responsiveness of T cells and suppress the presentation of tumor antigens by antigen-presenting cells [38]. When immunosuppression occurs, inflammation, measured with immunosuppressive leukocytes, plays a prognostic and possible tumor-promoting role [38]. However, neutrophils, the most important type of leukocyte, are associated with the development of glioma, particularly GBM [46]. A negative prognosis in GBM patients is associated with activated neutrophils, identified by surface expression of CD11b^+^ [46], but their recruitment and functional mechanisms are unknown. Several genes are able to recruit neutrophils, in particular immunosuppressive neutrophils at the tumor site, such as *IL8* which produced by glioma cells, factor associate suicide ligand (FasL) which was triggering, and MIF which was produced by glioma cancer stem cells [47–49]. Neutrophils which secrete elastase and produce S100A4 around the glioma could aid glioma infiltration and induce the proliferation of GBM-initiating cells [50, 51]. Our study revealed that the neutrophil-associated gene mentioned above is positively correlated with *PTRF* and caveolin-1 expression in glioma. Furthermore, neutrophils have been linked to the resistance to anti-angiogenic therapy in GBM [52]. It has been suggested that *PTRF* is involved in the signaling of immunological mediator molecules for the recruitment of immunosuppressive neutrophils and the promotion of glioma invasion and metastasis. The present study hypothesized that downregulating *PTRF* to decrease the number of caveolae and its signaling platform may weaken the immune response and stop the interaction between glioma and neutrophils, in particular the effect on certain immune-checkpoint pathways by glioma cells, and may lead to tumor regression. Therefore, more sophisticated approaches to investigate the immunology of *PTRF* functions are needed. We also found that *PTRF* is highly expressed in microvascular proliferation (MVP) regions and MES tumor cells, indicating that *PTRF* could involve in tumor microenvironment angiogenesis and drive transitions to MES-like states in gliomas. Finally, survival analysis revealed poor prognosis and short survival time in the group of patients with high expression of *PTRF* in their tumor sample. Therefore, *PTRF* may serve as a potential biomarker for early diagnosis and prognosis of high-grade glioma, and as a potential therapeutic target. Blockade of *PTRF*, which may reduce the number of caveolae, could prove to be an important tool in anti-glioma immunotherapy.

In conclusion, our study revealed that *PTRF* mRNA expression is associated with malignant progression and Genomic alterations in glioma, and plays an important role in tumor immune response; the expression of *PTRF* and caveolin-1 were found to be related to immune checkpoints, immunosuppression factors and pro-tumor activity in glioma. These findings suggest that high levels of *PTRF* predict worse survival for glioma patients. Finally, *PTRF* was associated with higher malignancy in glioma, which was inconsistent with the finding for other malignant tumors reported previously.

## Supplementary Information

**Additional file 1:** **Supplementary Figure 1.** Gene ontology functional enrichment maps from the CGGA dataset.

**Additional file 2:** **Supplementary Figure 2.** Gene ontology functional enrichment maps from the TCGA dataset.

## Data Availability

The datasets presented in this study can be found in online repositories (http://cancergenome.nih.gov and http://www.cgga.org.cn) ﻿, and the other data used to support the findings of this study are available from the corresponding author upon request.
