# Supplementary figures and images for: Molecular and clinical characterization of PTRF in glioma via 1,022 samples
Source: BMC Cancer. 2023 Jun 16;23:551. doi: 10.1186/s12885-023-11001-2 (PMC10273567; doi:10.1186/s12885-023-11001-2)

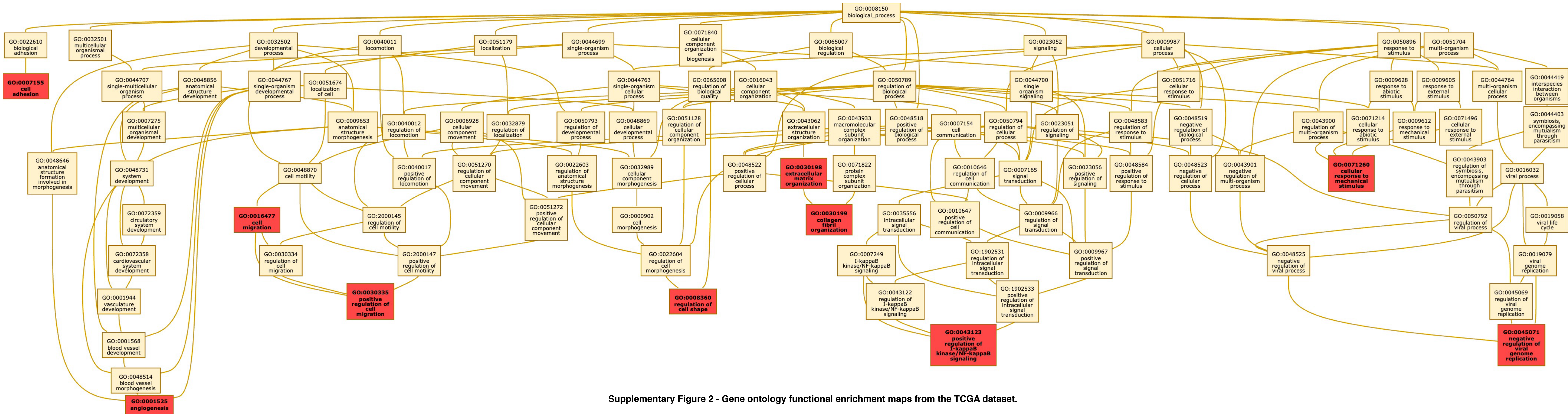

Supplement: Supplementary file 2 — Additional file 2: Supplementary Figure 2. Gene ontology functional enrichment maps from the TCGA dataset. [file 12885_2023_11001_MOESM2_ESM.pdf]
