# Supplementary material for: Molecular and clinical characterization of PTRF in glioma via 1,022 samples
Source: BMC Cancer. 2023 Jun 16;23:551. doi: 10.1186/s12885-023-11001-2 (PMC10273567; doi:10.1186/s12885-023-11001-2)
Supplement: Supplementary file 1 — Additional file 1: Supplementary Figure 1. Gene ontology functional enrichment maps from the CGGA dataset. [file 12885_2023_11001_MOESM1_ESM.pdf]

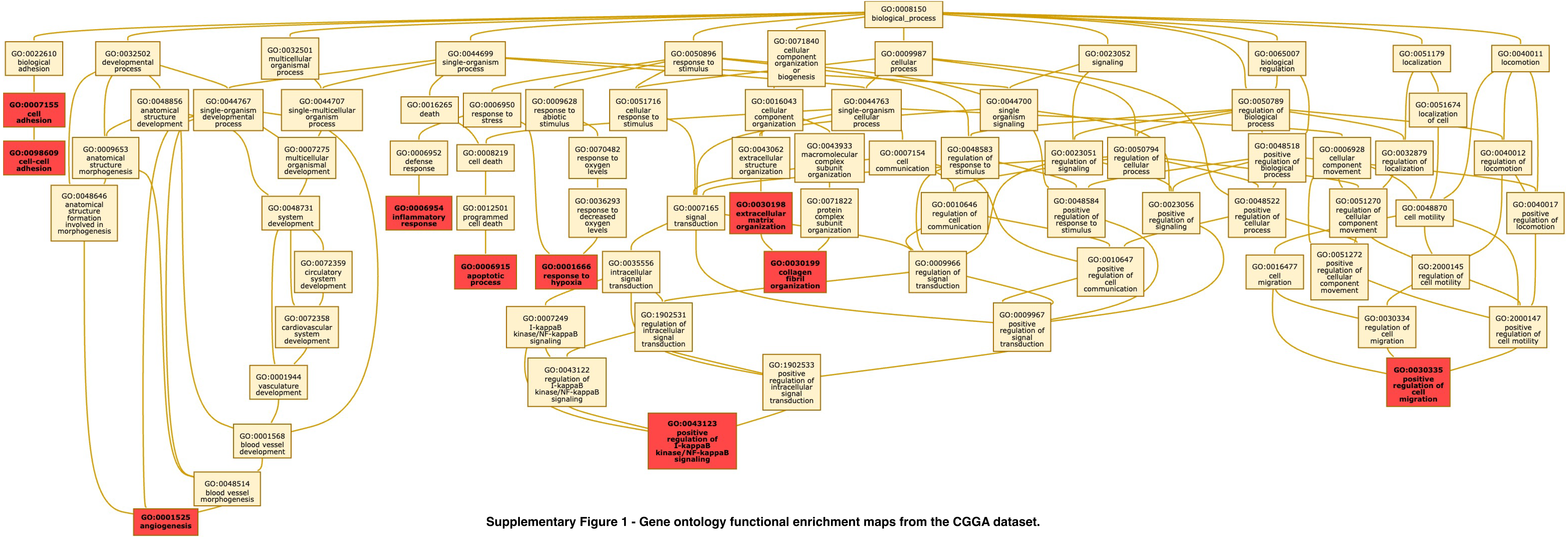

Supplementary Figure 1 - Gene ontology functional enrichment maps from the CGGA dataset.
